# Demographic characteristics and risk factors related to high blood pressure among healthy blood donors from Luanda, Angola: A retrospective study

**DOI:** 10.1002/hsr2.1300

**Published:** 2023-06-07

**Authors:** Cruz S. Sebastião, Euclides Sacomboio, Ngiambudulu M. Francisco, Joana Paixão, Edson K. Cassinela, Jocelyne Neto de Vasconcelos, Victor Pimentel, Joana Morais

**Affiliations:** ^1^ Instituto Nacional de Investigação em Saúde (INIS) Luanda Angola; ^2^ Centro de Investigação em Saúde de Angola (CISA) Caxito Angola; ^3^ Instituto de Ciências da Saúde (ICISA) Luanda Angola; ^4^ Global Health and Tropical Medicine (GHTM), Instituto de Higiene e Medicina Tropical (IHMT) Lisbon Portugal; ^5^ Centro de Estudos, Investigação Cientifica e Pós‐graduação (CEIP) Luanda Angola; ^6^ Centro Nacional de Investigação Científica (CNIC) Luanda Angola

**Keywords:** ABO/Rh blood groups, Angola, blood donors, demographic characteristics, hypertension, Luanda

## Abstract

**Background and Aims:**

Hypertension is a public health concern, mainly in resource‐limited countries. We investigated the characteristics and risk factors related to high blood pressure in healthy blood donors from, Luanda, the capital city of Angola.

**Methods:**

This was a retrospective study that included 343 healthy donors from December 2019 to September 2020.

**Results:**

The mean age was 32 ± 9 years. Men represented 93% of the population. Mean systolic blood pressure (SBP) was 131 ± 12.3 mmHg (ranging from 100 to 160 mmHg) and diastolic blood pressure (DBP) was 80.1 ± 9.72 mmHg (from 56.0 to 100 mmHg). DBP was related to age and gender (*p* < 0.05). About 7.3% of the donors had high‐pressure (>140/90 mmHg). Age between 20 and 40 years (odds ratio [OR]: 2.52, *p* = 0.043), women (OR: 1.87, *p* = 0.548), nonurbanized areas (OR: 0.39, *p* = 0.067), high educational level (OR: 0.76, *p* = 0.637), employed (OR: 0.49, *p* = 0.491), voluntary donors (OR: 0.87, *p* = 0.799), blood group B (OR: 2.06, *p* = 0.346), and Rh‐ (OR: 0.26, *p* = 0.104), were potentially related with high‐pressure. The high‐pressure cases increased from December 2019 (4%) to September 2020 (28%) (*p* = 0.019).

**Conclusion:**

We showed high pressure among the healthy blood donors population. Demographic characteristics, ABO/Rh blood group, and year period are features that should be considered in cardiovascular disease control strategies. Biological and nonbiological features related to blood pressure changes should be considered for further studies in the Angolan population.

## 1 INTRODUCTION

High blood pressure is one of the greatest global public health concerns affecting approximately one billion individuals worldwide^1^ and constitutes the main risk factor for coronary heart disease, heart failure, cerebrovascular disease as well as chronic renal failure,^2^, ^3^, ^4^ besides being responsible for about 30% of deaths worldwide annually.^5^, ^6^

The World Health Organization (WHO) estimated that low‐ and middle‐income countries (LMICs) have 93% of the world's disease burden and contributed to about 78% of deaths from cardiovascular disease and more than 50% of the total disease burden.^7^ If nothing is done to reduce the risk of chronic diseases in resource‐limited countries, an economic production estimated at US$ 84 billion will be lost to heart disease, stroke, kidney disease, and diabetes.^8^ This finding showed that high blood pressure is an essential feature of the epidemiological transition, although some studies have shown that there are no significant differences in the mean prevalence of hypertension between developed and developing countries.^9^, ^10^ However, it is worth mentioning here that the health profile of many LMICs, such as Angola, is undergoing major changes due to diet and lifestyle changes,^11^ therefore, with the increase in life expectancy diseases of old age are also increasing, including hypertension and cardiovascular disease, as previously described the relationship between old age and cardiovascular disease.^12^ These findings show that it is necessary to identify which biological or nonbiological characteristics might be influencing the emergence of healthy people with early diagnosis of high blood pressure, mainly in LMICs.

Since the 1990s, evidence has grown on the relative importance of systolic or diastolic blood pressure alone and together, on cardiovascular events.^13^ Currently, there are no published studies that describe the mean systolic or diastolic blood pressure, in the healthy population over 18 years of age in Angola. To the best of our knowledge, voluntary blood donors are the source of all red cell, platelet, and unprocessed plasma products used in clinical medicine.^14^ Therefore, in this study, we investigated the putative biological and nonbiological features that could affect blood pressure levels in the healthy population in Luanda, the capital city of Angola. The results of this study might be used by decision‐makers to reinforce ongoing practice guidelines^15^ for the management of arterial hypertension and cardiovascular diseases in Angola.

## 2 MATERIALS AND METHODS

### 2.1 Study design and setting

This was a retrospective multicentre cohort study that included 343 subjects who were assessed as healthy for blood donation at the Instituto Nacional de Sangue and Clínica Girassol, both health units located in Luanda, the capital city of Angola, between December 2019 to September 2020. All blood donor candidates who tested negative for transfusion‐transmitted infections (TTIs), such as human immunodeficiency virus, hepatitis B virus, hepatitis C virus, and syphilis, were included in the study, regardless of age or gender. The study was carried out at Instituto Nacional de Investigação em Saúde (INIS), also located in Luanda. The INIS is a public institution of the Angolan Ministry of Health (MoH), which develops research in the most diverse areas of health and its determinants, to contribute to the strengthening of public health policies in Angola.

### 2.2 Data collection and description

A structured questionnaire was used to collect sociodemographic information such as age, gender, place of residence, level of education completed, occupation, category of the donor, the period in which blood was donated, and ABO/Rh blood group. ABO/Rh blood group phenotypes determination was carried out using blood reagents and diagnostic kits (Lorne Laboratories Limited), following the manufacturer's instructions.^16^ Also, we used a Trucare KD‐558BR arm blood pressure monitor (Andon Health Co., Ltd.) for the measurement of systolic and diastolic blood pressure.^17^ To obtain more accurate measurements, blood donation candidates rested for at least 20 min before measuring blood pressure. The entire blood pressure measurement procedure, as well as the interpretation of systolic or diastolic blood pressure results, were carried out following the instructions provided by the manufacturer.^17^ High systolic blood pressure was considered when the values were greater than 140 mmHg, while diastolic blood pressure was considered high when the values were greater than 90 mmHg. For this study, we considered the Centers for Disease Control and Prevention guidelines and findings from previous studies, which show that a blood donation candidate can only be approved when at least the maximum systolic blood pressure is below 140 mmHg and the diastolic below 90 mmHg.^12^, ^15^, ^18^ However, candidates who had high blood pressure above 140/90 mmHg were grouped into the high or abnormal blood pressure category.

### 2.3 Statistical analysis

The analysis was conducted in SPSS version 28 (IBM SPSS Statistics). The descriptive analysis was presented as frequencies and percentages. The normal data distribution was presented as mean and standard deviation (SD). Parametric tests such as independent‐sample *t* tests and one‐way analysis of variance (ANOVA), were used to compare the mean values. Whenever possible, the variables were dichotomized and analyzed with the *χ* ^2^ test and logistic regression with a corresponding 95% confidence interval (CI) to predict features with a relation or independent chance to present high blood pressure. The reported *p* value is two‐tailed and was deemed statistically significant when *p* < 0.05.

## 3 RESULTS

### 3.1 Demographic characteristics related to high blood pressure

The putative demographic characteristic related to high blood pressure among blood donors from Luanda is summarized in Table 1. A total of 343 blood donors were eligible for donation and enrolled in the analyses. Age ranged from 18 to 61 years. The mean age of the blood donors was 32 ± 9 years old. Blood donors aged 20–40 years (81.3%), men (93%), living in nonurbanized areas (62.4%), highly educated blood donors (76.8%), employees (92.4%), and family blood donors (85.7%), were prevalent in this studied population.

**Table 1:** Demographic characteristics related to high blood pressure among blood donors from Luanda, Angola.

		Blood pressure index		Blood pressure status			Univariate analysis	
Demographic characteristics	*N* (%)	SBP (mmHg) Mean ± SD	DBP (mmHg) Mean ± SD	Abnormal (%)	Normal (%)	*p* Value^a^	OR (95% CI)	*p* Value
Overall	343 (100)	131 ± 12.3	80.1 ± 9.72	25 (7.30)	318 (92.7)			
Age groups (years)								
<20	7 (2.00)	130 ± 15.2	84.6 ± 6.97	0 (0.0)	7 (2.20)	0.083	‐	‐
20–40	279 (81.3)	130 ± 12.6	79.2 ± 9.81	17 (68.0)	262 (82.4)		2.52 (1.03–6.15)	**0.043**
>40	57 (16.6)	134 ± 13.8	84.1 ± 8.43	8 (32.0)	49 (15.4)		1.00	
*p* Value^b^		0.059	**<0.001**					
Gender								
Female	24 (7.00)	127 ± 11.0	75.5 ± 9.54	1 (4.00)	23 (7.20)	0.542	1.87 (0.24–14.5)	0.548
Male	319 (93.0)	131 ± 12.3	80.5 ± 9.66	24 (96.0)	295 (92.8)		1.00	
*p* Value^c^		0.090	**0.020**					
Residence area								
Nonurban	214 (62.4)	131 ± 12.3	80.3 ± 9.95	20 (80.0)	194 (61.0)	0.059	0.39 (0.14–1.07)	0.067
Urban	129 (37.6)	131 ± 12.3	79.8 ± 9.36	5 (20.0)	124 (39.0)		1.00	
*p* Value^c^		0.957	0.660					
Educational level^d^								
Low	59 (23.2)	134 ± 11.8	81.6 ± 10.9	4 (19.0)	55 (23.6)	0.636	1.00	
High	195 (76.8)	131 ± 13.0	80.8 ± 9.52	17 (81.0)	178 (76.4)		0.76 (0.25–2.36)	0.637
*p* Value^c^		0.118	0.586					
Occupation								
Unemployed	26 (7.60)	133 ± 11.9	80.0 ± 10.1	1 (4.00)	25 (7.90)	0.482	1.00	
Employed	317 (92.4)	131 ± 12.3	80.2 ± 9.70	24 (96.0)	293 (92.1)		0.49 (0.06–3.76)	0.491
*p* Value^c^		0.353	0.532					
Donor category								
Voluntary	49 (14.3)	130 ± 13.6	80.5 ± 9.25	4 (16.0)	45 (14.2)	0.799	0.87 (0.28–2.64)	0.799
Family	294 (85.7)	131 ± 12.1	80.1 ± 9.81	21 (84.0)	273 (85.8)		1.00	
*p* Value^c^		0.493	0.791					
Months 2019–2020								
December/2019	25 (7.3)	130 ± 11.7	78.9 ± 9.13	1 (4.00)	24 (7.50)	0.434	3.50 (0.41–30.1)	0.254
January/2020	145 (42.3)	130 ± 12.3	77.7 ± 8.59	8 (32.0)	137 (43.1)		2.50 (0.86–7.25)	0.093
February/2020	42 (12.2)	129 ± 12.2	78.6 ± 9.30	2 (8.00)	40 (12.6)		2.92 (0.57–14.8)	0.197
July/2020	52 (15.2)	131 ± 12.5	84.0 ± 10.5	4 (16.0)	48 (15.1)		1.75 (0.48–6.37)	0.396
August/2020	24 (7.0)	131 ± 13.6	82.9 ± 11.4	3 (12.0)	21 (6.60)		1.02 (0.48–6.37)	0.978
September/2020	55 (16.0)	136 ± 11.1	83.3 ± 9.67	7 (28.0)	48 (15.1)		1.00	
*p* Value^b^		**0.026**	**<0.001**					

*Note*: Abnormal: systolic > 140 or diastolic > 90 mmHg; Normal: systolic up to 140 mmHg or diastolic up to 90 mmHg. The bold numbers were statistically significant (*p* < 0.05).

Abbreviations: CI, confidence interval; DBP, diastolic blood pressure; OR, odds ratio; SBP, systolic blood pressure.

a *χ* ^2^.

^b^ One‐way analysis of variance.

^c^ Independent‐sample *t* tests.

^d^ Missing values: 89.

The overall mean systolic blood pressure index was 131 ± 12.3 mmHg (ranging from 100 to 160 mmHg), while the diastolic blood pressure index was 80.1 ± 9.72 mmHg (ranging from 56.0 to 100 mmHg). Systolic index values were higher among donors aged over 40 years, males, blood donors with a low educational level, unemployed, and familiar donors. On the other hand, diastolic index values were high among blood donors aged below 20 years, males, donors from nonurbanized areas, donors with low educational levels, employed, and voluntary donors. No statistically significant differences were observed in the mean values of systolic blood pressure and different demographic characteristics (*p* > 0.05). Regarding diastolic blood pressure, statistically significant differences were observed in the mean values for age (*p* < 0.001) and gender (*p* = 0.020).

Overall, 7.3% (25/343) of the blood donors had high blood pressure index, with systolic pressure > 140 mmHg and diastolic pressure > 90 mmHg. No statistically significant differences were observed between abnormal or normal blood pressure with the demographic characteristics (*p* > 0.05). About 68% of blood donors with abnormal blood pressure were aged between 20 and 40 years and this group was also the group most likely of having high blood pressure [odds ratio, OR: 2.52 (95% CI: 1.03–6.15), *p* = 0.043]. Men predominated in the group of donors with abnormal blood pressure with 96%. On the other hand, women even being the group with a low rate of high blood pressure cases (4%), were the group that presented the high odds of developing high blood pressure [OR: 1.87 (95% CI: 0.24–14.5), *p* = 0.548]. Donors residing in nonurbanized areas predominated the group of donors with abnormal blood pressure (80%), compared to donors residing in urbanized areas (20%). Despite this difference, the results of the logistic regression showed that the chances of developing high blood pressure are lower in the group of donors residing in nonurbanized areas [OR: 0.39 (95% CI: 0.14–1.07), *p* = 0.067]. Blood donors with a high educational level predominated in terms of the presence of high blood pressure with 81%. Despite that, the likelihood of developing high blood pressure was lower in blood donors with a high level of education [OR: 0.76 (95% CI: 0.25–2.36), *p* = 0.637], compared to the other group. Regarding the occupation of donors, we observed that donors who reported having some employment, were those who had more high blood pressure (96%), although logistic regression analyses showed that this group has a lower risk of developing high blood pressure [OR: 0.49 (95% CI: 0.06–3.76), *p* = 0.491], compared to unemployed blood donors. As for the type of donors, family donors predominated the group of donors with high blood pressure by about 84%. Our results show that voluntary blood donors, in addition to being less frequent about the presence of high blood pressure, are also a group with a slightly lower risk of developing high blood pressure [OR: 0.87 (95% CI: 0.28–2.64), *p* = 0.799].

From December 2019 to September 2020, the systolic (from 130 ± 11.7 to 136 ± 11.1 mm/Hg, *p* = 0.026) and diastolic (from 78.9 ± 9.13 to 83.3 ± 9.67 mm/Hg, *p* < 0.001) blood pressure indexes increased significantly. Moreover, we observed a significant increase in the rate of blood donors with high blood pressure increased significantly (*p* = 0.019) from 4% in December 2019 to 28% in September 2020. A higher rate of high blood pressure in blood donors was observed in January 2020 (32%, 8/25). Also, during this period blood donors were 2.5 times (95% CI: 0.86–7.25, *p* = 0.093) more likely to develop high blood pressure.

### 3.2 ABO/RH blood group and high blood pressure

The relationship between ABO/Rh blood groups with high blood pressure among blood donors from Luanda is shown in Table 2. Blood group O (63.6%, 218/343) and a positive Rh factor (97.4%, 334/343), were the most frequent. According to ABO/Rh blood group ABO/Rh, ORh+ (61.8%), ARh+ (16.9%), and BRh+ (15.7%) were the most frequent, while ABRh+ (2.90%), ORh− (1.70%), and ARh− (0.90%), the less frequent in this studied population. It is also worth mentioning that the blood groups BRh− and ABRh−, were not identified. There were no significant differences between the mean systolic or diastolic blood pressure values with ABO/Rh blood groups (*p* > 0.05). Also, no statistically significant relationship was observed between abnormal blood pressure and ABO/Rh blood groups (*p* > 0.05). The highest mean values of systolic (136 ± 12.2 mmHg) and diastolic (81.7 ± 11.3 mmHg) blood pressure were observed in the AB blood group, simultaneously. Despite that, no donor with blood group AB was part of the group with high blood pressure. On the other hand, blood group B (8%, 2/25) despite being the group with the lowest number of donors with high blood pressure, was the group with the highest chance of developing high blood pressure [OR: 2.06 (95% CI: 0.46−9.24), *p* = 0.346], compared with the other groups, although the results were not significant (*p* > 0.05).

**Table 2:** Relationship between ABO/RH blood group and high blood pressure among blood donors from Luanda, Angola.

		Blood pressure index		Blood pressure status			Univariate analysis	
Blood group distribution	*N* (%)	SBP (mmHg) Mean ± SD	DBP (mmHg) Mean ± SD	Abnormal (%)	Normal (%)	*p* Value^a^	OR (95% CI)	*p* Value
Overall	343 (100)	131 ± 12.3	80.1 ± 9.72	25 (7.30)	318 (92.7)			
ABO blood group								
A	61 (17.8)	132 ± 12.4	81.6 ± 9.28	7 (28.0)	54 (17.0)	0.334	0.61 (0.24–1.56)	0.303
B	54 (15.7)	132 ± 12.2	78.9 ± 9.86	2 (8.00)	52 (16.4)		2.06 (0.46–9.24)	0.346
AB	10 (2.90)	136 ± 12.2	81.7 ± 11.3	0 (0.00)	10 (3.10)		‐	‐
O	218 (63.6)	130 ± 12.3	79.9 ± 9.75	16 (64.0)	202 (63.5)		1.00	
*p* Value^b^		0.246	0.450					
RH blood group								
Rh−	9 (2.60)	137 ± 14.5	82.9 ± 14.7	2 (8.00)	7 (2.20)	0.081	0.26 (0.05–1.32)	0.104
Rh+	334 (97.4)	130 ± 12.2	80.0 ± 9.57	23 (92.0)	311 (97.8)		1.00	
*p* Value^c^		0.097	0.385					
ABO/RH blood group								
ARh+	58 (16.9)	132 ± 12.3	81.0 ± 8.92	6 (24.0)	52 (16.4)	0.267	1.00	
ARh−	3 (0.90)	137 ± 15.3	92.7 ± 11.0	1 (4.00)	2 (0.60)		0.23 (0.02–2.94)	0.259
BRh+	54 (15.7)	132 ± 12.2	78.9 ± 9.86	2 (8.00)	52 (16.4)		3.00 (0.58–15.6)	0.191
ABRh+	10 (2.90)	136 ± 12.2	81.7 ± 11.3	0 (0.00)	10 (3.10)		‐	‐
ORh+	212 (61.8)	130 ± 12.1	80.0 ± 9.62	15 (60.0)	197 (61.9)		1.52 (0.56–4.10)	0.413
ORh−	6 (1.70)	138 ± 15.5	78.0 ± 14.5	1 (4.00)	5 (1.60)		0.58 (0.06–5.80)	0.640
*p* Value^b^		0.203	0.222					

*Note*: Abnormal: systolic > 140 mmHg or diastolic > 90 mmHg; Normal: systolic up to 140 mHg or diastolic up to 90 mmHg.

Abbreviations: CI, confidence interval; DBP, diastolic blood pressure; OR, odds ratio; SBP, systolic blood pressure.

a *χ* ^2^.

^b^ Independent‐sample *t* tests.

^c^ One‐way analysis of variance.

About 92% of blood donors with abnormal blood pressure had Rh+ factor. The highest mean values of systolic (137 ± 14.5 mmHg) and diastolic (82.9 ± 14.7 mmHg) blood pressure were also observed in the Rh− factor, simultaneously, despite being the group with the lowest chance of developing high blood pressure [OR: 0.26 (95% CI: 0.05–1.32), *p* = 0.104]. Overall, blood donors in ORh− blood group were those with the highest mean values for systolic (138 ± 15.5 mmHg) blood pressure, while ARh− blood donors were the ones who presented higher diastolic (92.7 ± 11.0 mmHg) blood pressure. The blood groups that predominated for the presentation of high blood pressure were ORh+ (60%), ARh+ (24%), and BRh+ (8%), while the ORh− [OR: 0.58 (95% CI: 0.06–5.80), *p* = 0.640] and ARh− [OR: 0.23 (95% CI: 0.02–2.94), *p* = 0.259] blood groups were the least predominant with 4%, respectively and with the lowest chance of developing abnormal blood pressure. The BRh+ [OR: 3.00 (95% CI: 0.58–15.6), *p* = 0.191] and ORh+ [OR: 1.52 (95% CI: 0.56–4.10), *p* = 0.413] blood groups, were the more likely to develop abnormal blood pressure.

## 4 DISCUSSION

To the best of our knowledge, this was the first study to present in detail the putative features as well as risk factors for developing high blood pressure in a healthy population of Angola. Also, this is the first study that presents the possible mean values of systolic and diastolic blood pressure in the healthy general population, residing in Luanda, Angola. The prevalence of hypertension worldwide is approximately 26% of the adult population,^10^ which was higher compared to the observed in this study (7.3%). Epigenetic, sociodemographic, behavioral, and eating features of the Angolan or black population, could explain the low prevalence of about 3.6 times less hypertension in the Angolan population compared to global prevalence. According to our findings, about 7% of the healthy population in Luanda might have high blood pressure with a risk to develop cardiovascular disease,^4^ suggesting that further studies should be carried out to help control blood pressure in the healthy population of Angola.

Previous studies showed that the blood donation candidate can only be approved when at least the maximum systolic blood pressure is below 140 mmHg and the diastolic below 90 mmHg.^12^, ^15^, ^18^ The mean values of systolic (131 ± 12.3 mmHg) and diastolic (80.1 ± 9.72 mmHg) pressure in our study correspond with the global blood pressure norms for the healthy population (Table 1). However, it is worth mentioning that the maximum systolic blood pressure was 160 mmHg and the maximum diastolic blood pressure was 100 mmHg, suggesting that some blood donors from Angola, a sub‐Saharan African country, are experiencing high blood pressure and risk of developing stroke, myocardial infarction, heart and/or renal failure,^2^, ^3^, ^4^ even before donating at least 450 mL of whole blood. Previous studies carried out independently contributed to a universal discovery in which reducing blood pressure levels in the general population remarkably reduces cardiovascular morbidity and mortality, as well as slowing the progression of kidney disease, retinopathy, and death from all causes.^1^, ^19^ Therefore, further studies assessing changes in blood pressure levels pre‐ and postblood donation should be carried out among blood donors, to help in the detection of possible cardiac complications pre‐ and postblood donation, as well as identify and indicate early treatment for individuals with the predisposition of developing cardiovascular disease.

The sociodemographic description of this study, highlighting the predominance of young donors aged up to 40 years old, male, residents in nonurbanized regions, and with some employment is in agreement with population‐based studies carried out in Angola,^11^ as well as other studies indicating that the Angolan population is young, living in undeveloped huts, and with limited sanitary access.^20^, ^21^, ^22^ Regarding the relationship between age and blood pressure, previous studies have shown that systolic blood pressure tends to increase with age, while diastolic blood pressure decrease.^12^, ^13^ This physiological pattern was confirmed by our results in the blood donors population from Luanda, as we identified a significant increase in mean of systolic blood pressure (from 130 ± 15.2 to 134 ± 13.8 mmHg, *p* = 0.059) and a slight decrease in mean of diastolic blood pressure (from 84.6 ± 6.97 to 84.1 ± 8.43 mmHg, *p* < 0.001) with increasing age from under 20 years to over 40 years, respectively (Table 1).

We expected that the highly educated population in our study would be more likely to develop high blood pressure since the increase in the level of education would also imply increasing age, which is an important risk factor for hypertension.^12^, ^13^ Interestingly, blood donors with a high educational level had 0.76 times less likely to develop high blood pressure (Table 1), contradicting the results of a study carried out in Brazil in which 72% of the hypertensive population had a high educational level.^5^ In addition, this study carried out in Brazil reported a significant relationship (*p* = 0.004) between low income and the presence of hypertension, where the authors observed that 65.8% of the hypertensive population had the lowest salary.^5^ Our results are in agreement with these findings since we observed that the donors employed were 0.49 times less likely to develop high blood pressure, compared to the unemployed population. Indeed, the lack of employment, lack of resources with the high cost of living checked around the world are factors that could affect blood pressure levels in the population, mainly the population from LMICs, although a recent study reported that there are no significant differences in the prevalence of hypertension between developed and developing countries.^9^ On the other hand, despite the females being predominant in Angola, representing about 52% of the general population,^11^ only 7% were part of this study (Table 1). Our results are in agreement with the findings reported by Sahu et al., in a study carried out with blood donors from India, where was reported also a low adherence of the female gender (1.4%) compared to the male gender (98.6%).^23^ Even without statistical significance (*p* > 0.05), our findings indicate that women are 1.87 times more likely to develop high blood pressure compared to men. Interestingly, the female gender has already been identified as a group prone to developing cardiovascular disease in Angola. In a recent study carried out by our research team, including patients with arterial hypertension and undergoing treatment in a tertiary unit in Luanda, we found that about 73% (72/99) of the hypertensive patients were female.^24^ Our results also correspond to a study carried out by Silva et al., with hypertensive patients in the state of São Paulo, Brazil, where 62.1% of the hypertensive population were female.^5^ Furthermore, a study comparing the hypertensive population of Portugal and immigrants from the portuguese speaking African countries, revealed that 51% and 67% of the population in both groups were hypertensive women from Portugal and immigrants, respectively.^25^ The physiological aspects such as hormonal climacteric changes, menopause, menstruation, pregnancy, and breastfeeding inherent to the female gender could explain the reduced participation of females in blood donation as well as being a factor for present high blood pressure.

The large adhesion of blood donors during January 2020 is not surprising, and the possible explanation is that once in this period, Luanda province like other Angola provinces, face heavy rainfall, and due to poor sanitation, there is an increase in stagnant water and thus increases also the circulation of vectors capable of increasing cases of malaria, dengue, or other vector‐borne diseases in Luanda, as verified by other studies.^22^, ^26^, ^27^, ^28^, ^29^ However, the increase in malaria and dengue cases could be one of the reasons for the increase in blood donation since many patients with malaria or dengue need blood transfusions during hospital treatment.^30^ Indeed, this explanation could be supported by our results, since the risk of having high blood pressure in January 2020 was 2.5 times, compared to the other months of the same year. Also, we observed that the mean value of systolic (from 130 ± 11.7 to 136 ± 11.1 mmHg, *p* = 0.026) and diastolic (from 78.9 ± 9.13 to 83.3 ± 9.67 mm/Hg, *p* < 0.001) blood pressure increased significantly from December 2019 to September 2020 (Table 1). Interestingly, the first cases of SARS‐CoV‐2 infection in Angola were identified in January 2020, by our research team.^21^ In the present study, we showed that the blood pressure values as well as the frequency (from 4% in December 2019 to 28% in September 2020, *p* = 0.434) of the healthy population residing in Luanda, have increased with the spread time of the endemic form of the SARS‐CoV‐2 in Angola. We do not know whether this increase in blood pressure was influenced by previous exposure to SARS‐CoV‐2. Therefore, further studies evaluating the blood pressure pattern in an Angolan population exposed to SARS‐CoV‐2 infection should be carried out in the future. It is worth mentioning that other reasons, such as the pressure to donate blood due to a family member who needs a blood component transfusion, could also cause an increase in blood pressure levels. Indeed, our results showed that family donors (85.7%, 294/343) have been predominant in the blood donor population and this was also the group in which we find about 84% of individuals with high blood pressure. As we already expected, volunteer donors had 0.87 times less risk of developing abnormal blood pressure, possibly justified by the fact that this group not having any family, medical or social pressure to make a blood donation.

A previous study reported that the Angolan general population is mostly from the blood group O and Rh+,^21^, ^31^, ^32^ which is in agreement with the findings of our study (Table 2). Despite this, our results disagree with the results of a recent study carried out in 2020 by Sacomboio et al., where they identified a higher prevalence of blood group B (36.4%, *n* = 36/99) in a population from Luanda,^24^ instead of the O group. We cannot rule out the fact that in recent years, there may be an increase in the Angolan population with group B while reducing the population with group O, especially in the population with chronic diseases. From the point of view of evolution and trend of ABO blood groups in the general population between 1973 and 2020, there was an increase in blood group O (from 52% to 64%) and a decrease in blood groups A (from 21% to 18%) and B (from 24% to 16%), while the AB blood group remained around 3%.^31^ On the other hand, comparing the evolution within the hypertensive population in recent years (from 2019 to 2020) according to ABO blood group phenotypes, there was an increase in blood pressure among individuals of the groups O (from 33% to 64%) and A (from 17% to 28%) and a reduction among individuals of the group B (from 36% to 8%) and AB (from 13% to 0%).^24^ Despite the reduction of blood group B in the general and hypertensive population over the years, we identify that group B donors had up to three times more risk of developing high blood pressure, corresponding with the findings of Sacomboio et al., which revealed that blood group B individuals, seem to be more susceptible to hypertension.^32^ In this study, we did not explore the reasons why blood group B individuals present a higher risk of developing high blood pressure, despite the decline in frequency in the Angolan population. Therefore, further studies assessing the possible biological or nonbiological factors that could influence systole or diastole blood pressure values in the general population, especially those belonging to blood group B, must be carried out.

This study has some potential limitations. Firstly, the small sample size of participants limited the significance of our results and may not be representative of the whole Angolan population. However, to obtain an updated and more representative picture of the hypertension situation in the healthy Angolan population, it is important to gather the most recent data and quantify the rate of high blood pressure in the healthy population from the different regions of Angola. Second, a family history of hypertension or chronic disease, especially in the donor population with high blood pressure, was not obtained or described in this studied population. Finally, a follow‐up and assessment of blood pressure after blood donation or clinical outcome (recovered or deceased) whether from the blood donor after blood donation or the patient after receiving the blood component, was not performed on all blood donors included in this study, which limits the retention of the strong conclusion if these donors were hypertensive or the high blood pressure values were due to a clinical or psychosocial condition. Despite these weaknesses, our findings provide an important description of the mean values of systolic and diastolic blood pressure in the healthy population from Angola. Therefore, must be carried out further studies including epigenetic, sociodemographic, behavioral, clinical, and eating features, as well as, a description of the clinical reasons for carrying out the donation in the case of family donors. Also, studies assessing blood pressure postblood donation over some time including laboratory tests to assess cardiac, hepatic, or renal function, should be considered for future investigation. These studies could provide a specific picture of which population should adhere more to the primary prevention measures of hypertension, involving actions at the community level such as reducing obesity, consumption of alcohol and salt as well as increased physical activity.^15^

## 5 CONCLUSION

We showed high levels of systolic and diastolic blood pressure in the healthy population of Luanda, Angola. Individuals under 40 years of age, men, living in nonurbanized areas, with a high level of education, with some occupation, and family donors represented a risk group to develop hypertension. Blood group O was the most predominant, despite blood group B representing a high‐risk group for developing hypertension and possibly cardiovascular disease. Further studies assessing biological and nonbiological features related to blood pressure changes should be carried out in the Angolan population.

## AUTHOR CONTRIBUTIONS

**Cruz S. Sebastião**: Conceptualization; data curation; formal analysis; investigation; project administration; supervision; validation; writing—original draft; writing—review and editing. **Euclides Sacomboio**: Data curation; formal analysis; investigation; writing—review and editing. **Ngiambudulu M. Francisco**: Investigation; writing—review and editing. **Joana paixão**: investigation; project administration; writing—review and editing. **Edson K. Cassinela**: Investigation; writing—review and editing. **Jocelyne Neto Vasconcelos**: Investigation; project administration; writing—review and editing. **Victor Pimentel**: Data curation; formal analysis; writing—review and editing. **Joana Morais**: Investigation; project administration; writing—review and editing.

## CONFLICT OF INTEREST STATEMENT

The authors declare no conflict of interest.

## ETHICS STATEMENT

This study was approved by the National Ethics Committee of the Angolan MoH (no. 10/2021), the direction board of the Instituto Nacional de Sangue (no. 726/GDG/INS/2020), and the executive committee of the Clínica Girassol (no. 0841/GEPP/PCE/2021). Being a retrospective study, informed consent was waived by the National Ethics Committee of the Angolan MoH.

## TRANSPARENCY STATEMENT

The lead author Cruz S. Sebastião affirms that this manuscript is an honest, accurate, and transparent account of the study being reported; that no important aspects of the study have been omitted; and that any discrepancies from the study as planned (and, if relevant, registered) have been explained.

## Data Availability

All relevant data are within the article.
